# Modulation of Pineal Melatonin Synthesis by Glutamate Involves Paracrine Interactions between Pinealocytes and Astrocytes through NF-**κ**B Activation

**DOI:** 10.1155/2013/618432

**Published:** 2013-08-05

**Authors:** Darine Villela, Victoria Fairbanks Atherino, Larissa de Sá Lima, Anderson Augusto Moutinho, Fernanda Gaspar do Amaral, Rafael Peres, Thais Martins de Lima, Andréa da Silva Torrão, José Cipolla-Neto, Cristóforo Scavone, Solange Castro Afeche

**Affiliations:** ^1^Laboratory of Pharmacology, Butantan Institute, Avenida Vital Brasil 1500, 05503-900 São Paulo, SP, Brazil; ^2^Department of Physiology and Biophysics, Institute of Biomedical Sciences, University of São Paulo Avenida Professor Lineu Prestes 1524, 05508-900 São Paulo, SP, Brazil; ^3^Department of Pharmacology, Institute of Biomedical Sciences, University of São Paulo, Avenida Professor Lineu Prestes 1524, 05508-900 São Paulo, SP, Brazil

## Abstract

The glutamatergic modulation of melatonin synthesis is well known, along with the importance of astrocytes in mediating glutamatergic signaling in the central nervous system. Pinealocytes and astrocytes are the main cell types in the pineal gland. The objective of this work was to investigate the interactions between astrocytes and pinealocytes as a part of the glutamate inhibitory effect on melatonin synthesis. Rat pinealocytes isolated or in coculture with astrocytes were incubated with glutamate in the presence of norepinephrine, and the melatonin content, was quantified. The expression of glutamate receptors, the intracellular calcium content and the NF-**κ**B activation were analyzed in astrocytes and pinealocytes. TNF-**α**'s possible mediation of the effect of glutamate was also investigated. The results showed that glutamate's inhibitory effect on melatonin synthesis involves interactions between astrocytes and pinealocytes, possibly through the release of TNF-**α**. Moreover, the activation of the astrocytic NF-**κ**B seems to be a necessary step. In astrocytes and pinealocytes, AMPA, NMDA, and group I metabotropic glutamate receptors were observed, as well as the intracellular calcium elevation. In conclusion, there is evidence that the modulation of melatonin synthesis by glutamate involves paracrine interactions between pinealocytes and astrocytes through the activation of the astrocytic NF-**κ**B transcription factor and possibly by subsequent TNF-**α** release.

## 1. Introduction

The mammalian pineal gland is a component of the photoneuroendocrine system and is involved in the regulation of circadian rhythms through the nocturnal synthesis of melatonin. The gland consists mainly of pinealocytes but also presents interstitial cells, the majority of which are astrocytes [1, 2]. The pinealocytes are the neuroendocrine cells that synthesize melatonin and secrete it into the bloodstream. In mammals, this process is controlled by the retinohypothalamic pathway and sympathetic innervation, with norepinephrine being released in the perivascular space of the gland at night [3–5]. Norepinephrine interacts with its proper *β*1 and *α*1 receptors and stimulates the transcription and translation of the key enzyme arylalkylamine N-acetyltransferase, AANAT (EC 2.3.1.87), resulting in melatonin synthesis and secretion [6–10]. The role of astrocytes on melatonin production, however, remains unclear.

Several neurotransmitters can modulate melatonin synthesis [10–12]. Acetylcholine and glutamate are two important modulators that inhibit melatonin synthesis by decreasing the gene expression and activity profile of both AANAT and hydroxyindole-O-methyltransferase (HIOMT) (EC 2.1.1.4). Glutamate is stored in synaptic-like microvesicles in pinealocytes and is released upon cholinergic stimulation [13, 14]. The interaction of glutamate with AMPA-type ionotropic receptors in the pinealocytes induces greater glutamate exocytosis, amplifying the cholinergic action [15, 16]. Recently, an investigation demonstrated that the glutamate vesicular transporter could be responsible for pinealocyte depolarization and glutamate release [17]. The final effect of glutamate results from its binding to mGluR3, a class II metabotropic glutamate receptor, which is coupled to the inhibitory G-protein (Gi), leading to reductions in the amount of cAMP and the synthesis of melatonin [12]. 

Studies have shown the presence of many types of glutamate receptors in the pineal gland. In addition to the known AMPA and mGluR3 (40, 42) expression in the pineal gland, Yatsushiro and colleagues showed that rat pinealocytes express mGluR5 [18], a class I metabotropic receptor related to inositol triphosphate (IP_3_) formation and intracellular calcium mobilization, whose role on pineal metabolism has not yet been defined. The presence of glutamate receptors in pineal astrocytes has also been demonstrated. NMDA and AMPA ionotropic receptors were characterized in the rat pineal gland [19], while mGluR2, mGluR3, and mGluR5 were identified in the gerbil pineal gland [20]. These data may indicate that glutamatergic signaling in the pineal gland is more complex than previously thought and involves both astrocytes and pinealocytes, similar to the pattern observed in the central nervous system (CNS).

Indeed, in the CNS, astrocytes are important partners at the synapses. Over the past decade, an increasing number of observations have progressively challenged the classical view that astrocytes are passive participants in synaptic function. In fact, there is a dynamic two-way communication between glia and neurons, called tripartite synapse, in which astrocytic processes are associated with the presynaptic and postsynaptic neuronal elements [21]. Neurotransmitters released by presynaptic neurons evoke a calcium increase in adjacent astrocytes which in turn can release several transmitters, called gliotransmitters, including glutamate, D-serine, ATP, and large molecules like atrial natriuretic factor and tumor necrosis factor-*α* (TNF-*α*) [21–24]. These gliotransmitters feed back onto the presynaptic neuron either to enhance or to depress further release of the neurotransmitters [25], or they can act on postsynaptic neurons, causing excitatory or inhibitory responses [26]. 

Glutamate signaling in the CNS is classically related to the activation of nuclear factor *κ*B (NF-*κ*B). Thus, glutamate induces the transcription of genes such as nitric oxide synthase, cyclooxygenase 2, Mn-superoxide dismutase, calbindin, glutamate receptors, Bcl-2, and brain-derived neurotrophic factor [27–29]. Additionally, recent studies have shown that astrocytes use NF-*κ*B as a mediator of transcriptional responses to a variety of stimuli, including tissue injury and diseases [30]. NF-*κ*B can induce or repress gene expression by binding to DNA sequences known as *κ*B elements [31, 32]. Activation of NF-*κ*B typically involves the phosphorylation of proteins that inhibit NF-*κ*B (I*κ*Bs) by the I*κ*B kinase (IKK) complex, which results in I*κ*B degradation [33]. NF-*κ*B is then released from the inhibitory complex and translocated to the nucleus. In mammalian cells, there are five NF-*κ*B family members—Rel A (p65), Rel B, c-Rel, p50/p105 (NF-*κ*B1), and p52/p100 (NF-*κ*B2)—and different NF-*κ*B complexes are formed by diverse homo- and heterodimerization processes [34, 35]. In the pineal gland, two independent studies showed that NF-*κ*B is involved in the inflammatory response and exhibits a circadian rhythm [36, 37]. 

Based on these data, we aimed to investigate the inhibitory action of glutamate on melatonin synthesis, analyzing the crosstalk between astrocytes and pinealocytes. NF-*κ*B activation, glutamate receptor expression, and intracellular calcium responses were analyzed in both cell types. In the present work, we demonstrated that the paracrine interactions between pinealocytes and astrocytes are fundamental for the glutamate inhibitory effect on melatonin synthesis and that it involves the activation of astrocytic NF-*κ*B transcription factor and the release of a soluble factor, possibly TNF-*α*.

## 2. Materials and Methods

### 2.1. Animals

Male Wistar rats (200–220 g) were kept under a 12 : 12 h light-dark cycle (lights on at 06 : 00 h) in a temperature-controlled room (21 ± 2°C) with water and food *ad libitum*. The animals were euthanized by decapitation between 09 : 00 and 10 : 00 h. All experiments were performed in accordance with the guidelines of the Brazilian College for Animal Experimentation (COBEA) and approved by the Committee of Ethics in Animal Experimentation of the Institute of Biomedical Sciences (CEUA—Permit number: 091), University of São Paulo (São Paulo, Brazil).

### 2.2. Pineal Gland Culture

Pineal glands were cultured as previously described [38]. Briefly, after decapitation, the rat pineal glands were removed and immediately placed in ice-cold BGJb medium (Fitton-Jackson Modification, Gibco, Grand Island, NY, USA) with phenol red, modified by the addition of bovine serum albumin (1 mg/mL), 2 mM glutamine, 0.1 mg/mL ascorbic acid, and penicillin (100 U/mL)-streptomycin (100 *μ*g/mL). Pineal glands were cultivated (37°C, 95% O_2_, 5% CO_2_) in the same BGJb medium in 24-well plates (2 glands/well; 200 *μ*L/well) for 48 h (the medium was changed after the first 24 h). After this period, all glands were placed in fresh medium for 1 h before treatment. For melatonin analysis, the glands were incubated with a combination of the following drugs: norepinephrine (NE) (1**** *μ*M), glutamate (100 and 600**** *μ*M), and pyrrolidine dithiocarbamate (PDTC) (300 *μ*M). After 5 h, the glands were frozen on dry ice and kept at −80°C until they were assayed. All culture media components and drugs used were purchased from Sigma (St. Louis, MO, USA).

### 2.3. Isolated Pinealocyte Culture

Pinealocytes were obtained by papain digestion (Papain Dissociation System, Worthington Biochemical Corporation, Freehold, NJ, USA). The glands were removed and immediately placed in ice-cold Dulbecco's Modified Eagle's Medium (DMEM) (glucose: 1000 mg/L, HEPES: 5.9 g, and sodium bicarbonate: 3.7 g) (Sigma, St. Louis, MO, USA). The tissue was incubated at 37°C for 45 min in papain (0.01%) and DNase (0.01%) solution. After removal of papain and its blockage with ovomucoid (2 mg/mL), the cells were mechanically dispersed and resuspended in DMEM supplemented with 10% fetal calf serum and 1% penicillin-streptomycin to a final concentration of 2 × 10^5^ cells/mL. Fifteen milliliters of cells in culture medium was cultivated in 75 cm^2^ culture flasks at 37°C in 5% CO_2_/95% air for 24 h. After 24 h, the cells in suspension were predominantly pinealocytes, which were separated after the removal of all the culture medium content. The cells were centrifuged and resuspended in DMEM at a concentration of 2 × 10^5^ cells/mL, transferred to a 24-well plate (1 mL per well), and kept at 37°C in 5% CO_2_/95% air for 1 h prior to the pharmacological treatments. For melatonin determination in the culture medium, the cells were stimulated with NE (1**** *μ*M) alone or in association with glutamate (100 or 600**** *μ*M) for 5 h.

### 2.4. Isolated Astrocyte Culture

After the removal of the pinealocytes in suspension, the astrocytes remained attached to the culture flasks and were kept in culture for one week. After this period, the cells were washed twice with Hanks solution, and 6 mL of trypsin 0.25% was added for 5 min. Trypsin action was blocked with DMEM supplemented with 10% fetal calf serum. The cells were centrifuged at 300 g for 5 min at 20°C. The pellet was resuspended according to the different assays.

### 2.5. Pinealocyte and Astrocyte Coculture

After the dissociation of the pineal gland cells by papain digestion, pinealocytes and astrocytes were cocultivated in the same well (24-well plates) in DMEM medium with 10% fetal calf serum at 37°C in 5% CO_2_/95% air at a concentration of 4 × 10^5^ cells/mL (1 mL per well) for 60 h. After that, the medium was replaced by fresh medium without fetal calf serum, and the cells were kept for 1 h at 37°C in 5% CO_2_/95% air prior to the pharmacological treatments. For melatonin determination in the culture medium, the cells were stimulated with NE (1**** *μ*M) alone or in association with glutamate (100 or 600**** *μ*M) for 5 h. When BB1101 (10**** *μ*M) [39], the inhibitor of TNF-*α* converting enzyme (TACE; ADAM17), was used, it was added to the medium 30 min before the treatments described previously.

The cells in co-culture were also physically separated by inserts (transwell with 0.4 *μ*m pore; Corning, NY, USA), with astrocytes in the bottom of the wells and pinealocytes within the inserts. The incubation procedures described previously were reproduced.

### 2.6. Cell Culture Characterization by Immunocytochemistry

Suspensions of pineal gland cultured cells (astrocytes, pinealocytes, or co-culture) were washed twice in phosphate buffer (PB, 0.1 M, pH 7.4) for medium removal and fixed with 2% paraformaldehyde in PB for 15 min. After two additional washes in PB, cells were spread upon gelatin- and chromoalumen-coated slides and allowed to dry and adhere using a hot plate (37°C). Cells were then incubated with mouse monoclonal antibodies either against the glial fibrillary acidic protein (GFAP; Sigma, USA) or CR3 complement receptor (OX-42, BD Biosciences, USA), diluted 1 : 1000–1 : 2000 in PB containing 0.01% Triton X-100 in a wet chamber for 14–18 h at room temperature. Following 3 × 10 min washes in PB, cells were then incubated with a wheat germ agglutinin-rhodamine label (WGA; Vector Laboratories, USA) mixed with a donkey anti-mouse IgG conjugated to fluorescein isothiocyanate (Jackson Labs, USA), diluted 1 : 200 and 1 : 100, respectively, for 2 h. Slides were then washed 3 × 10 min and coverslipped with glycerol-carbonate buffer. The material was analyzed using a fluorescence microscope equipped with standard filters, and digital images were collected and mounted using Adobe Photoshop software (Adobe Systems Inc., USA). Controls for the experiments consisted of the omission of primary antibodies or agglutinin from the procedure. No staining was observed in these cases.

### 2.7. Intracellular Calcium Measurement

Isolated astrocytes and pinealocytes were obtained as described previously. The cells were placed on a thin glass coverslip and incubated in DMEM with 16 nM of Fluo-4 (Molecular Probes Inc., Eugene, OR, USA), kept at 37°C for 1 h, and then washed three times with the same medium. Isolated astrocytes and pinealocytes were treated with glutamate (600**** *μ*M) or glutamatergic agonists (N-methyl-D-aspartic acid—NMDA 100**** *μ*M; *α*-amino-3-hydroxyl-5-methyl-4-isoxazole-propionate—AMPA 50**** *μ*M; (S)-3,5-dihydroxyphenylglycine—DHPG 50**** *μ*M) (Tocris Bioscience, Ellisville, MO, USA). A laser scanning microscope (LSM 510, Carl Zeiss, Jena, Germany) equipped with the Attofluor ratio imaging system (Atto Instruments, Rockville, MD, USA) was used. Measurements were performed using an excitation filter of 505 nm, and the emission was monitored at 530 nm.

### 2.8. Determination of Melatonin Content

Melatonin content in pineal glands and culture medium was determined by high performance liquid chromatography (HPLC) with electrochemical detection using Empower software (Waters System, Milford, MA, USA) [38]. Melatonin was separated on a resolve C18 column (5 *μ*m, 150 × 3.9 mm). The chromatographic system was isocratically operated with the following mobile phase: 0.1 M sodium acetate, 0.1 M citric acid, and 0.15 mM EDTA, 30% methanol, pH 3.7, at a flow rate of 1 mL/min. The electrochemical detector potential was adjusted to +900 mV. The elution time for melatonin was approximately 6 min. Each gland was sonicated (Microson XL 2005, Heat System Inc., Farmingdale, NY, USA) in a solution of 0.1 M perchloric acid, containing 0.02% EDTA and 0.02% sodium bisulfate. Culture medium was diluted in the same solution of perchloric acid (1 : 1 vol/vol). After centrifugation (2 min, 13,000 g), 40 *μ*L of the supernatant was injected into the chromatographic system (Injector Mod. 7125, 20 *μ*L loop, Rheodyne Inc., San Francisco, CA, USA). All the salts and reagents used were obtained from Merck (Frankfurter, Darmstadt, Germany).

### 2.9. Electrophoretic Mobility Shift Assay

#### 2.9.1. Pineal Gland Nuclear Protein Extracts

Nuclear extracts were obtained from pineal gland homogenates as previously described [37] after incubation for 30 min with or without glutamate (600**** *μ*M). Briefly, the glands were homogenized in lysis buffer (10 mM HEPES, 10 mM KCl, 0.1 mM EDTA, 10% glycerol, and 0.1 mM PMSF), and after 15 min, nonyl phenoxypolyethoxylethanol (NP)-40 (10%) was added. The samples were vigorously mixed, centrifuged (12,000 g, 1 min, 4°C) twice, and resuspended in extraction buffer (10 mM HEPES, 0.5 M KCl, 1 mM EDTA, 10% glycerol, and 0.1 mM PMSF). After incubation on ice for 15 min and centrifugation (20,000 g, 5 min, 4°C), the supernatant containing the nuclear proteins was collected and stored at −80°C. Protein concentration was determined using the BioRad protein reagent (BioRad, Hercules, CA, USA).

#### 2.9.2. Astrocytes and Pinealocytes Nuclear Protein Extracts

Nuclear extracts were obtained from isolated astrocytes and pinealocytes homogenates as previously described [40] after incubation for 30 min with or without glutamate (600**** *μ*M). Briefly, the cells were homogenized in lysis buffer (10 mM HEPES, 1.5 mM MgCl_2_, 10 mM KCl, 0.5 mM PMSF, 0.1 mM EDTA, and 3 mM orthovanadate). After adding NP-40 (10%), samples were vigorously mixed, centrifuged (11,000 g, 20 min, 4°C) twice, and resuspended in extraction buffer (20 mM HEPES, 1.5 mM MgCl_2_, 300 mM NaCl, 0.25 mM EDTA, 25% glycerol, 0.5 mM PMSF, 3 mM orthovanadate, 2 *μ*g/mL leupeptin, and 2 *μ*g/mL antipain). After a 20 min incubation on ice and centrifugation (11,000 g, 20 min, 4°C), the supernatant containing the nuclear proteins was collected and stored at −80°C. The protein concentration was determined using the BioRad protein reagent.

#### 2.9.3. Gel Shift Assay

An electrophoretic mobility shift assay for NF-*κ*B was performed utilizing the gel shift assay kit from Promega (Madison, WI, USA), as described previously [41]. A ^32^P-NF-*κ*B double-stranded consensus oligonucleotide probe (5′-AGTTGAGGGGACTTTCCCAGGC-3′) and nuclear extracts were used. DNA-protein complexes were separated by electrophoresis through a 6% nondenaturing acrylamide:bisacrylamide (37.5 : 1) gel in 0.53 Tris-borate-EDTA (TBE) for 2 h at 150 V. Gels were vacuum dried and analyzed by autoradiography. For competition experiments, NF-*κ*B and transcription initiation factor II (TFIID 5′-CAGAGCATATAAGGTGAGGTAGGA-3′) unlabeled double-stranded consensus oligonucleotide were included in ten-fold excess of the molar concentration of ^32^P-NF-*κ*B probe to detect specific and nonspecific DNA-protein interactions, respectively. Unlabeled oligonucleotides were added to the reaction mixture 20 min before the addition of the radioactive probe. The composition of the complexes was determined by supershift assays, adding antibodies (Santa Cruz Biotechnologies, Santa Cruz, CA, USA) against different NF-*κ*B subunits (p50, p52, c-Rel, and p65, 1 : 20 dilution) either before or after the incubation of nuclear extracts with the labeled oligonucleotide. 

### 2.10. RNA Extraction and RT-PCR

Total RNA was extracted from isolated astrocytes and pinealocytes using TRIzol (Invitrogen, Carlsbad, CA, USA) according to the manufacturer's instructions. cDNA synthesis was performed using SuperScript III Reverse Transcriptase (RT) (Invitrogen, Carlsbad, CA, USA) from 1 *μ*g of total RNA. After the RT reaction, 2-*μ*L aliquots were used as a cDNA template for polymerase chain reaction (PCR) amplifications using GoTaq DNA Polymerase (Promega, Madison, WI, USA). The cycling parameters used were 30 s at 95°C, 30 s at each primer's annealing temperature, and 30 s at 72°C using a MyGene Series Gradient Thermocycler (LongGene, China). PCR products were separated on 1.2% ethidium bromide-agarose gels, and the bands were visualized by digital scanning. The primers used in this assay are presented in supplementary Table 1 (see Table 1 in Supplementary Material available at http://dx.doi.org/10.1155/2013/618432).

### 2.11. Flow Cytometry

Pineal glands in culture were stimulated with NE 1**** *μ*M in the absence or presence of glutamate 100**** *μ*M or 600**** *μ*M. After 5 h of incubation, the cells were dissociated via proteolytic digestion with papain. In an assay of membrane integrity, 50 *μ*L of a propidium iodide solution (20 *μ*g per mL in saline buffer) was added to the cells (500 *μ*L). Propidium iodide is a highly water-soluble fluorescent compound that cannot pass through intact membranes, as it is unable to enter viable cells. After membrane disruption, it binds to DNA by intercalating between the bases with little or no sequence preference. After 5 min of incubation at room temperature, the cells were evaluated in FACScalibur flow cytometer equipment (Becton Dickinson, San Jose, CA, USA) using Cell Quest software (Becton Dickinson, San Jose, CA, USA). Fluorescence was measured using the FL2 channel (orange-red fluorescence—585/42 nm). DNA fragmentation was analyzed after DNA staining with propidium iodide according to the previously described method [42]. Cells were resuspended in a solution with detergents (300 *μ*L hypotonic solution containing 20 *μ*g/mL propidium iodide, 0.1% sodium citrate, and 0.1% Triton X-100) that permeabilize the cells, which promptly incorporate the dye into DNA. The pellet was gently resuspended, and the cells were then incubated for 2 h at room temperature. Fluorescence was measured and analyzed by flow cytometry as described previously.

### 2.12. Statistical Analysis

Data were always presented as the mean ± SEM. Statistical analyses (GraphPad Prism 5.0 software, San Diego, CA, USA) were performed using one-way ANOVA followed by a Bonferroni post hoc test. When appropriate, Student's *t*-test was applied. The graphs related to confocal microscopy were prepared utilizing Origin 5.0 software (MicroCal Software Inc., Northampton, MA, USA). Autoradiographs of NF-*κ*B were quantified using ScionImage software (Scion Corp., Frederick, MD, USA).

## 3. Results

**Figure 1:**
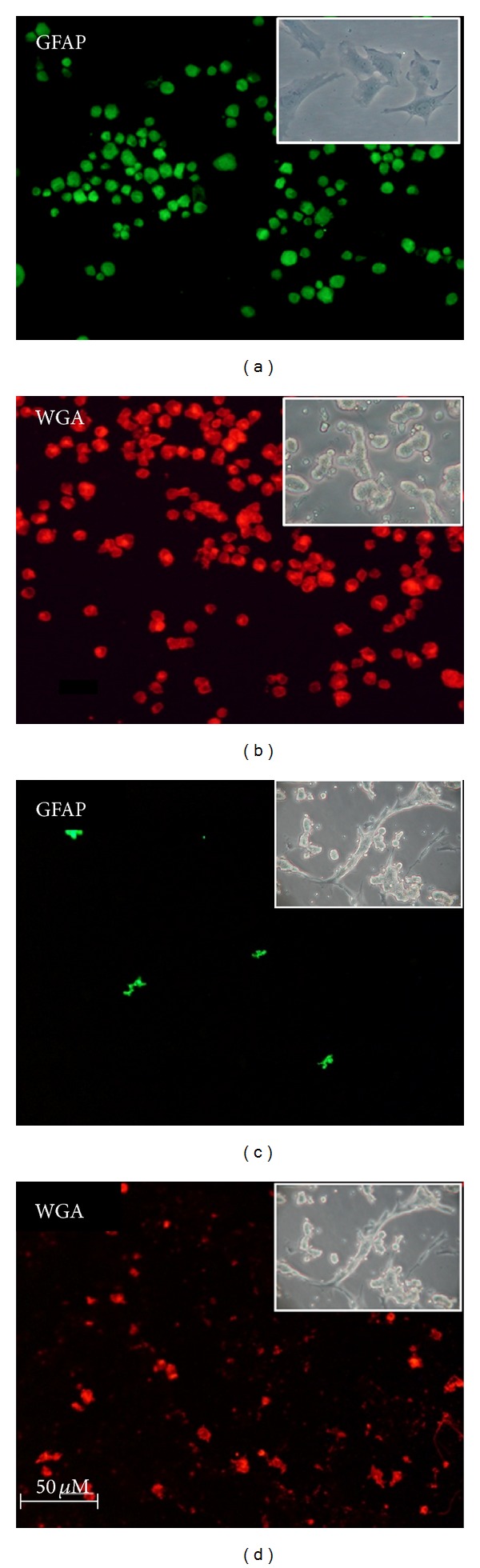
Cell cultures immunocytochemistry. Digital images of isolated cultures of astrocytes (a) and pinealocytes (b) showing specific cell type staining using a mouse monoclonal antibody against the glial fibrillary acidic protein (GFAP) and a wheat germ agglutinin-rhodamine label (WGA) to identify pinealocytes. The pictures at the bottom illustrate the distribution of glial cells (c) and pinealocytes (d) in a mixed coculture. The insets show photomicrographies of the cell cultures in contrast phase microscopy: astrocytes (a), pinealocytes (b) and co-culture (c and d). In isolated pinealocytes and in co-culture, many cells are clustered. Magnification: 200x (astrocytes and pinealocytes) and 100x (co-culture).

The cultures used in this study were characterized and validated by immunostaining with GFAP, a specific marker for astroglial cells, and OX-42, a marker for microglia (specific for activated microglia, as the antibody reacts with the CR3 complement). This OX-42 antibody is suitable for pineal gland staining because the predominant type of microglia in this tissue expresses complement type 3 receptors [43]. WGA was used as a pinealocyte marker because it labels photoreceptors [44, 45], and pinealocytes are modified photoreceptor cells [46, 47]. This technique proved to be efficient in labeling these pineal cells (Figure 1). 

Cultured isolated astrocytes showed fluorescence for GFAP (Figure 1(a)) but not for OX-42 and WGA (data not shown). Cultured pinealocytes lacked immunostaining for the two glial markers GFAP and OX-42 (data not shown) and reacted with WGA, demonstrating the absolute predominance of pinealocytes (Figure 1(b)). Figures 1(c) and 1(d) show the distribution of pinealocytes and astrocytes in the mixed culture (co-culture). The round appearance of the labeled cells in Figure 1, different from that in the insets, is due to the fixation process with paraformaldehyde. In Figure 1(d), pinealocytes appear smaller than those in Figure 1(b), most likely due to the use of trypsin to obtain the cells, further dissociating the cell clusters.

**Figure 2:**
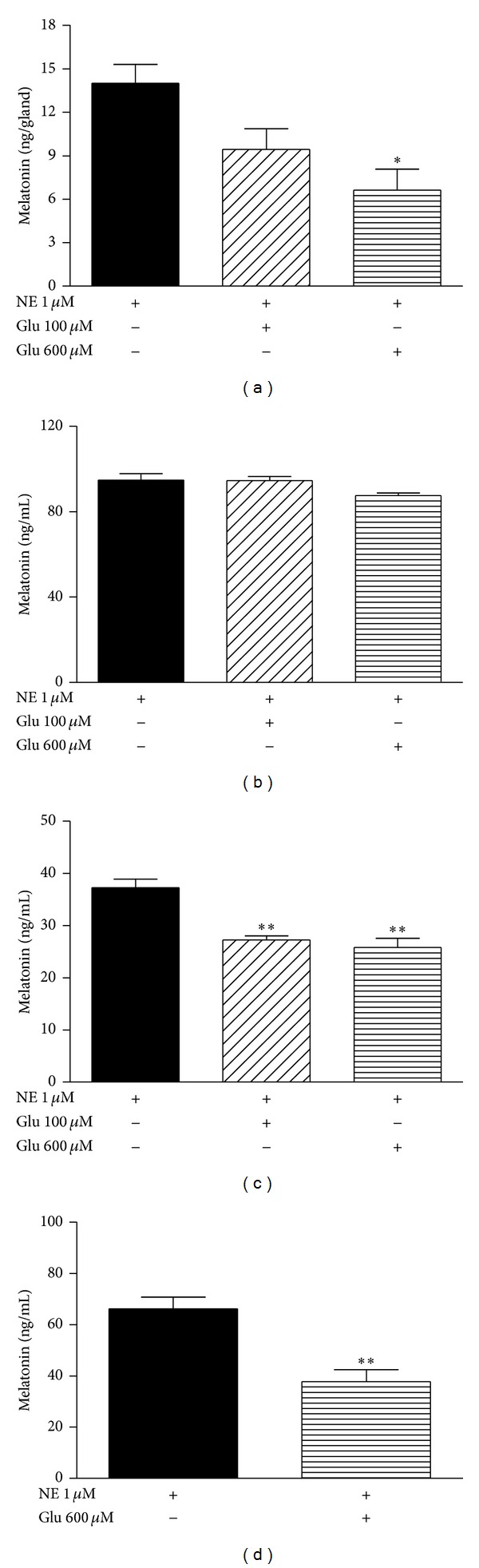
Glutamate effects on melatonin synthesis. (a) Pineal gland culture stimulated with norepinephrine (NE 1 *μ*M) in the absence or presence of glutamate (Glu 100 and Glu 600 *μ*M). Melatonin content is expressed in ng/gland as the mean ± SEM of 3 experiments. One-way ANOVA, Bonferroni multiple comparison test, *P < 0.05 versus NE 1 *μ*M, *n* = 12 for each group. (b) Isolated rat pinealocyte culture under NE 1 *μ*M stimulation in the absence or presence of Glu 100 or Glu 600 *μ*M. *n* = 6 for each group. (c) Pinealocyte and astrocyte co-culture stimulated with NE 1 *μ*M in the absence or presence of Glu 100 or Glu 600 *μ*M. One-way ANOVA, Bonferroni multiple comparison test, **P < 0.01 versus NE 1 *μ*M, *n* = 8 for each group. (d) Astrocytes and pinealocytes were cultured together but with physical separation by inserts. Astrocytes were cultivated in the bottom of the plate, and pinealocytes were cultivated within the inserts and stimulated with NE 1 *μ*M, associated or not associated with Glu 600 *μ*M. *T* test, **P < 0.01, *n* = 4 for each group. For the cell cultures, melatonin was evaluated in the culture medium and expressed in ng/mL as the mean ± SEM of 3 experiments.

In intact cultured pineal glands, the known effect of glutamate in reducing NE-stimulated melatonin synthesis (*P* < 0.05) was observed (Figure 2(a)). On the other hand, when isolated cultured pinealocytes were incubated with glutamate (100 and 600**** *μ*M) in the presence of NE (1**** *μ*M), this effect was abrogated (Figure 2(b)). Glutamate's inhibitory effect on melatonin synthesis, as observed in intact cultured pineal glands, was only reproduced when pinealocytes were cultivated in the presence of astrocytes in co-culture (*P* < 0.01) (Figure 2(c)).

To investigate if the contact between astrocytes and pinealocytes is necessary for the inhibitory effect of glutamate, the two cell types were cocultivated in the same well but separated by inserts. It was observed that glutamate's inhibitory effect on NE-stimulated melatonin synthesis was expressed in the same way as when the cells were cultivated with physical contact, indicating that a diffusible factor released by astrocytes acted on the pinealocytes (*P* < 0.01) (Figure 2(d)).

**Figure 3:**
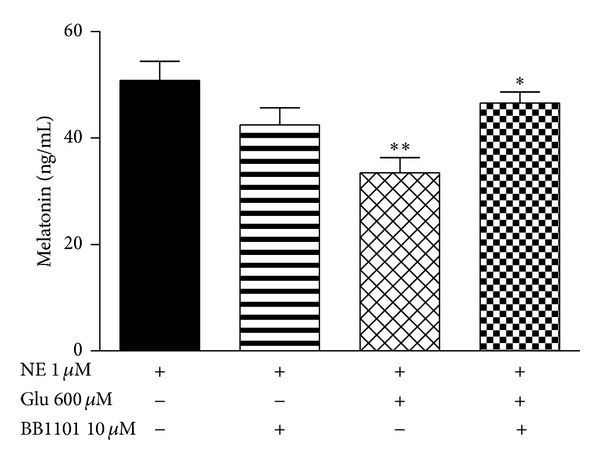
(a) Ablation of the inhibitory glutamatergic effect on melatonin synthesis by the TACE inhibitor BB1101. Pinealocytes and astrocytes were maintained in co-culture and stimulated with norepinephrine (NE 1 *μ*M) in association with glutamate (Glu 600 *μ*M) and BB1101 (10 *μ*M). One-way ANOVA, Bonferroni multiple comparison test, **P < 0.01 versus NE 1 *μ*M, *P < 0.05 versus NE 1 *μ*M + Glu 600 *μ*M, *n* = 4 for each group, 3 experiments.

The possibility of TNF-*α* as a gliotransmitter that mediates the glutamate effect was tested using BB1101, a preferred inhibitor of metalloproteinases with sheddase activity that includes the TACE. It was verified that when the cells in co-culture were incubated with BB1101 (10**** *μ*M) prior to the treatments with glutamate (600**** *μ*M) and NE (1**** *μ*M), it was able to completely revert the inhibitory effect of glutamate on melatonin synthesis (*P* < 0.05) (Figure 3).

**Figure 4:**
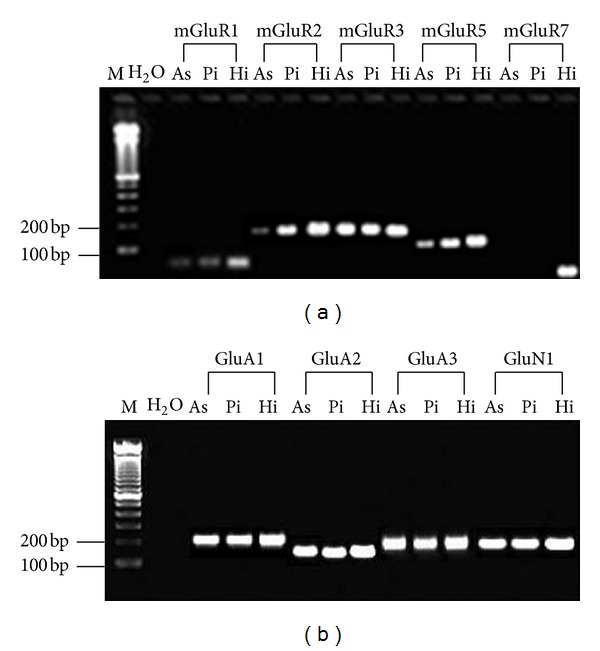
RT-PCR detection of metabotropic and ionotropic glutamate receptors in isolated astrocytes and pinealocytes. (a) RT-PCR detection of transcripts for mGluR1 (79 bp), mGluR2 (198 bp), mGluR3 (196 bp), mGluR5 (165 bp), and mGluR7 (70 bp) in astrocytes (As), pinealocytes (Pi), and hippocampus (Hi). Molecular weight marker (M) and water (H_2_O) are presented in lanes 1 and 2, respectively. (b) RT-PCR detection of transcripts for GluA1 (203 bp), GluA2 (155 bp), GluA3 (193 bp), and GluN1 (201 bp) in astrocytes (As), pinealocytes (Pi), and hippocampus (Hi). The molecular weight marker (M) and PCR product generated with water (H_2_O) are presented in lanes 1 and 2, respectively.

**Figure 5:**
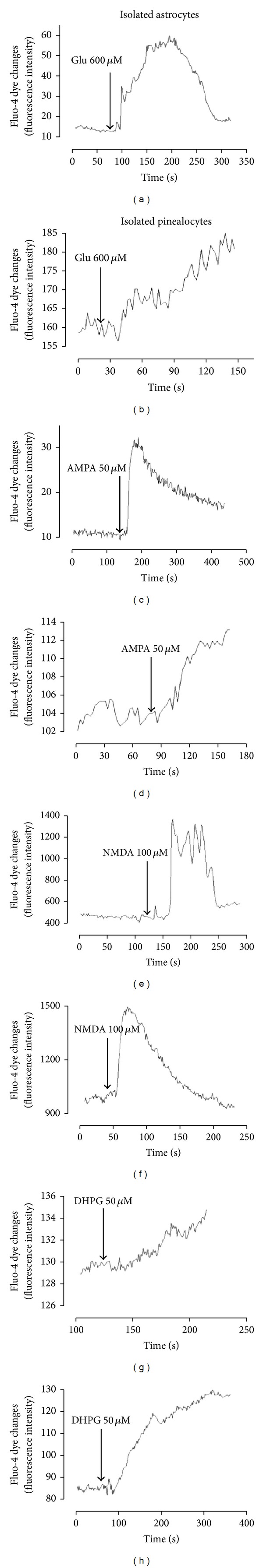
Analysis of intracellular calcium in isolated astrocyte and pinealocyte cultures. Traces of Fluo-4 fluorescence indicate the intracellular calcium transients from single cells. Additions of exogenous glutamate or glutamatergic agonists were performed as indicated by the arrows.

RT-PCR analyses showed mRNA expression of class I/II metabotropic glutamate receptors (mGluR1/mGluR5 and mGluR2/mGluR3, resp.), as well as ionotropic NMDA and AMPA-type receptors (GluN1, GluA1, GluA2, and GluA3 subunits) in astrocytes and pinealocytes (Figures 4(a) and 4(b)). Contrarily, mGluR7 mRNA (class III metabotropic receptors) was not detected in any of the cell types (Figure 4(a)). To determine whether class I mGluRs, NMDA, and AMPA ionotropic receptors are functional and incorporated into the astrocytic and pinealocytic membranes, an intracellular calcium analysis by confocal microscopy was used. It was observed that the class I metabotropic receptor agonist DHPG (50**** *μ*M) and the ionotropic receptor agonists NMDA (100**** *μ*M) and AMPA (50**** *μ*M) were able to increase the astrocytes and pinealocytes intracellular calcium content in a similar way as that observed for glutamate (600**** *μ*M) (Figure 5, Table 1). 

**Figure 6:**
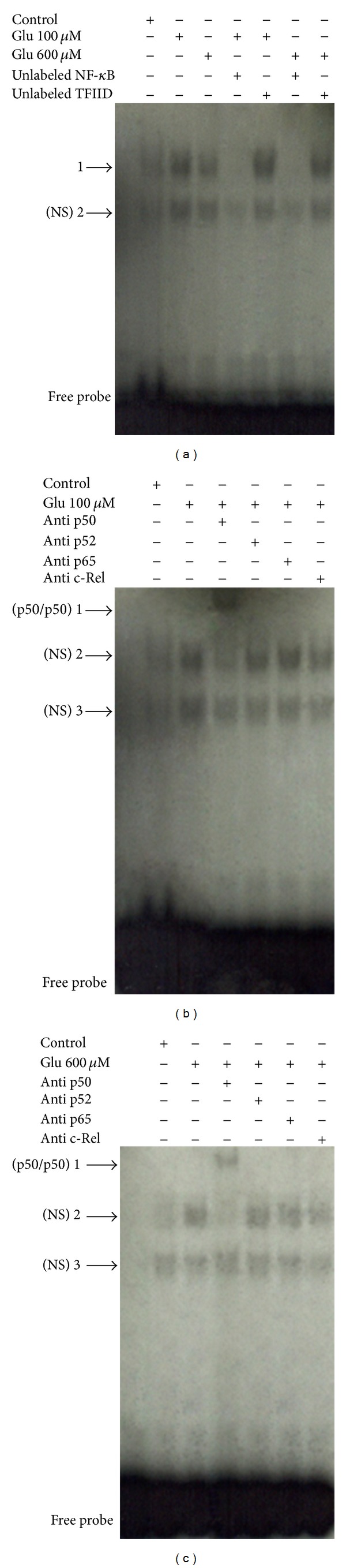
Electrophoretic mobility shift assays for NF*κ*B in the pineal gland. (a) Competition studies were performed using nuclear extract (5 *μ*g) from cultured pineal glands treated with glutamate (Glu) 100 and 600 *μ*M, in the absence or presence of unlabeled specific (NF-*κ*B consensus sequence, 10-fold molar excess) or nonspecific oligonucleotide (TFIID consensus sequence, 10-fold molar excess), as indicated. (b, c) Supershift assays were performed with the same nuclear extract (5 *μ*g) incubated in the absence or presence of antibodies against the subunits p50 (1 : 10 dilution), p65 (1 : 20 dilution), p52, and c-Rel (1 : 10 dilution), as indicated. Antibodies were added 20 min prior to the addition of the radiolabeled NF-*κ*B consensus oligonucleotide. The position of specific NF-*κ*B/DNA binding complexes p50/p50 (band 1, b, c) is indicated. NS represents no specific binding (bands 2 and 3, a, b, c). The localization of the free probe is also indicated.

Electrophoretic mobility shift assays revealed that glutamate (100 and 600**** *μ*M) activates NF-*κ*B in cultured rat pineal glands. The result of the competition study showed that the upper complex 1 was displaced by an excess of unlabeled NF-*κ*B but not by a TFIID double-stranded oligonucleotide consensus sequence, demonstrating the specificity of the NF-*κ*B/DNA interaction. The lower complex 2 was less efficiently displaced by unlabeled NF-*κ*B probe (Figure 6(a)). Supershift analysis indicated that the antibody against the p50 subunit was able to shift the DNA/protein interaction present in complex 1. The presence of antibodies against the p52, p65, and c-Rel subunits did not affect DNA-protein complexes (Figures 6(b) and 6(c)). Taken together, these results indicate that, at these concentrations, glutamate activates the p50/p50 homodimer. Complex 2 was not displaced by the antibodies and was not considered to be related to the NF-*κ*B family. 

**Figure 7:**
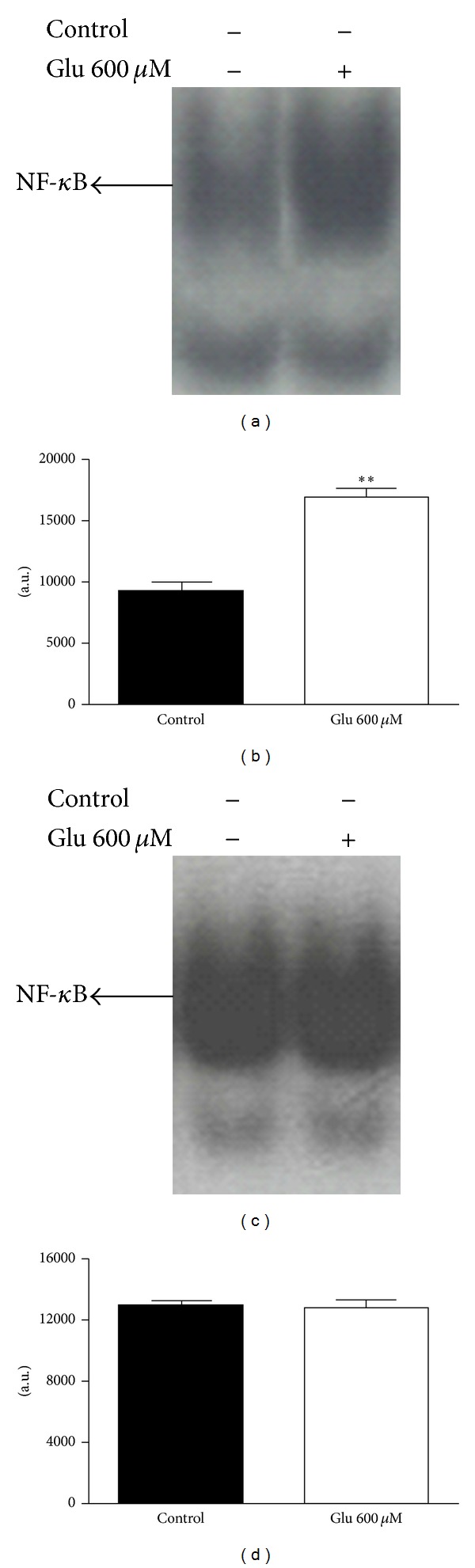
Electrophoretic mobility shift assays for NF*κ*B in isolated astrocytes and pinealocytes. (a) Nuclear proteins (5 *μ*g) were extracted from astrocytes treated with glutamate (Glu) 600 *μ*M. (b) Densitometric analysis (arbitrary units) of the NF-*κ*B band is presented on panel (a). One-way ANOVA, Student's test, **P < 0.01 versus control group, mean ± SEM. (c) Nuclear proteins (5 *μ*g) were extracted from pinealocytes treated with Glu 600 *μ*M. (d) Densitometric analysis (arbitrary units) of the NF-*κ*B band is presented in (c). The position of the specific NF-*κ*B/DNA binding complex is indicated. *n* = 5 for each group, 3 experiments.

Astrocyte and pinealocyte isolated cultures were used to determine the cell type in which NF-*κ*B was being activated. Glutamate (600**** *μ*M) was shown to activate NF-*κ*B exclusively in astrocytes and not in pinealocytes (*P* < 0.01) (Figure 7).

**Figure 8:**
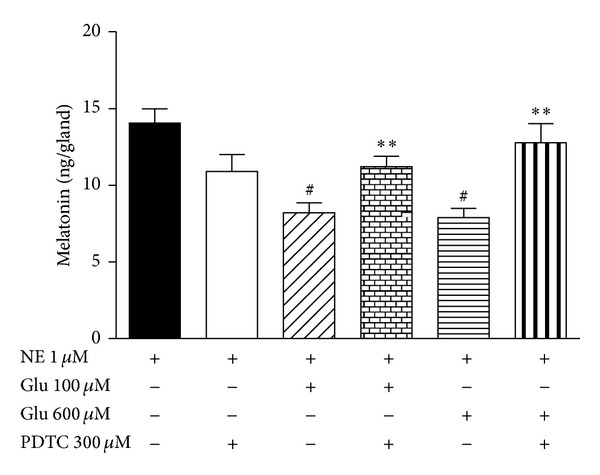
Effect of PDTC, an inhibitor of NF-*κ*B, on melatonin synthesis. Pineal glands in culture were incubated with PDTC (300**** *μ*M) and stimulated by norepinephrine (NE 1**** *μ*M) in the presence of glutamate (100 and 600**** *μ*M), and melatonin content was evaluated. One-way ANOVA, Bonferroni multiple comparison test, ^#^ *P* < 0.01 versus NE 1**** *μ*M, **P < 0.01 versus NE + Glu 100**** *μ*M and NE + Glu 600**** *μ*M, plotted as the mean ± SEM, *n* = 12 for each group, 3 experiments.

The involvement of NF-*κ*B activation in glutamate's inhibitory effect on melatonin synthesis was analyzed using a specific inhibitor of NF-*κ*B, PDTC. PDTC (300**** *μ*M) prevented the inhibitory effect of glutamate in cultured rat pineal glands stimulated by NE (1**** *μ*M) (*P* < 0.01) (Figure 8). 

Glutamate cytotoxicity was evaluated because it is known that this neurotransmitter in high concentrations could induce cell death by cytotoxicity [48]. Cell membrane integrity and DNA fragmentation were assessed by flow cytometric analysis using propidium iodide (PI). Supplementary Figure 1 is representative of the results obtained after cell incubation with NE (1**** *μ*M) in the absence (control group) or presence of glutamate (100 and 600**** *μ*M) for 5 h. The histograms show the fluorescence intensity in the FL-2 channel, which corresponds to the PI emission wavelength. When the cell membrane is intact, the fluorescence signal is low and is related to autofluorescence (M1). A high signal means that PI was able to enter the cell and bind to DNA (M2). Glutamate treatments for 5 h were not able to induce a loss of membrane integrity (Supplementary Figure 1(a)). The percentage of cells with ruptured membranes in the control group was 4.53%, whereas it was 5.02% and 5.77% in the 100 and 600**** *μ*M glutamate-treated groups, respectively, (Supplementary Figure 1(c)). Histograms in Supplementary Figure 1(b) represent the results of DNA fragmentation analysis. After PI treatment, the DNA content in live cells presented a high fluorescence signal (M2). DNA fragmentation induces the formation of small DNA pieces that exhibit low fluorescence intensity (M1). The percentages of cells with DNA fragmentation after glutamate stimulation, 100 and 600**** *μ*M, were 2.86% and 3.35%, respectively, which were similar to the value of control cells (3.66%) (Supplementary Figure 1(d)).

## 4. Discussion

Isolated pinealocytes, isolated astrocytes and pinealocyte/astrocyte co-culture were used in this study. The immunocytochemistry characterization of the cell types present in these different cultures demonstrated the predominance of pinealocytes in isolated pinealocyte culture, with the absence of glial cells. In the same way, astrocyte cultures do not contain a significant number of microglial cells or pinealocytes. In co-culture, pinealocytes are the predominant cells, as astrocytes are present in minor quantities, reproducing the proportion present in intact pineal glands (51). Thus, the cultures were considered to be appropriate for this study.

Data from the literature have shown that glutamate acts as an inhibitory neurotransmitter on melatonin synthesis [12]. According to Yamada et al. [13, 14, 16] and Yatsushiro et al. [16], glutamate is stored in microvesicles and released from pinealocytes upon cholinergic stimulation. It interacts with ionotropic and metabotropic glutamate receptors present in pinealocyte membranes, releasing additional glutamate and inhibiting melatonin synthesis, respectively. Recently, a mechanism involving the glutamate transporter GLT-1 was proposed to be responsible for glutamate release [17]. In these studies involving pinealocytes, an autocrine/paracrine process was suggested for the influence of glutamate on melatonin synthesis. In the present work, we demonstrated an important role for astrocytes in glutamate's modulatory effect on melatonin synthesis that includes a paracrine interaction with pinealocytes. 

Whereas in isolated pinealocytes, glutamate does not exhibit the typical inhibitory effect on melatonin synthesis, as observed in intact pineal glands; its association with astrocytes restores this effect. These results demonstrate that astrocytes act as partners that collaborate to produce the glutamate effect. In addition, the fact that the effect occurred even when inserts were used, preventing the physical contact between the cells in co-culture, suggests that a soluble factor is a mediator of this process. The hypothesis that gap junctions mediated the interactions between astrocytes and pinealocytes, as occurs in the pineal gland [49], was excluded from this observation. The cytokine TNF-*α*, a gliotransmitter that is released by astrocytes, could be the soluble factor that is involved in the interactions between astrocytes and pinealocytes. In fact, the absence of a soluble form of TNF-*α* interferes with the glutamate response.

The possibility that the glutamate effects were due to cell death was investigated by flow cytometry. As the cell viability and DNA fragmentation levels were similar between control and glutamate-stimulated groups, showing no damage to pineal cells even when treated with 600**** *μ*M of glutamate, it is possible to exclude a glutamate cytotoxic effect as a cause of melatonin reduction. 

Although glutamate receptor expression had been first described in pinealocytes, its expression was also later demonstrated in astrocytes. mGluR3 and AMPA (GluA1 subunit) were shown to be present in pinealocytes [12, 16], and NMDA and AMPA (GluA2/3) receptors were present in rat pineal gland astrocytes [19]. Ionotropic receptor mRNA was similarly observed in astrocytes in the present study, in addition to the novel observation of metabotropic receptor (mGluR1, mGluR2, mGluR3, mGluR5) expression. In pinealocytes, the expression of mGluR1/R2/R3/R5, AMPA, and NMDA receptors was shown. This evidence supports the possibility of astrocyte participation in rat pineal glutamatergic signaling. 

Confocal microscopy assays, using NMDA, AMPA, and DHPG agonists in isolated astrocytes and pinealocytes, revealed increased intracellular calcium content in both cell types for all the agonists used. PCR and confocal microscopy analyses suggest both gene and protein expression in class I mGluR, AMPA, and NMDA ionotropic receptors, revealing the importance of the glutamate receptors as signal transducers in rat pineal gland astrocytes and pinealocytes. Thus, glutamate seems to interact with its receptors in both cell types, but its effect on melatonin synthesis necessarily involves astrocyte glutamate receptors. The possible paracrine interactions between pinealocytes and astrocytes seem to be the key in understanding the role of astrocytes in the rat pineal gland.

Transcription factors exert a great influence on pineal physiology. CREB and ICER are two transcription factors that intimately control the transcription of AANAT [50]. Additionally, there are other transcription factors in the rat pineal gland whose roles in pineal melatonin synthesis are not known [51]. NF-*κ*B is present in the rat pineal gland, showing a circadian rhythm and involvement in inflammatory responses [36, 37]. NF-*κ*B is a classic possible glutamate mediator, and its activation in glial cells was demonstrated to play a role in inflammatory processes [30]. The present results demonstrated NF-*κ*B activation by glutamate in cultured isolated astrocytes, supporting its role as a mediator of glutamate effects. Supershift assay in cultured pineal glands stimulated with glutamate showed the displacement of the p50 subunit, emphasizing the importance of p50/p50 homodimer formation. This is an astrocyte-dependent event because its activation was not observed in pinealocytes. Astrocyte and pinealocyte supershift assays were not performed due to technical limitations related to the amount of nuclear protein.

The p50 subunit is synthesized from its precursor p105. The processing of p105 and the resulting generation of mature p50 subunits seem to be a constitutive event, as p50 homodimers are not regulated by I*κ*B proteins [34]. The p50 subunit formation seems to be regulated by cotranslational and posttranslational mechanisms. In the posttranslational mechanism, the p50 subunit is synthesized from its precursor p105 by phosphorylation at its 927 and 932 serine residues, which allows the ubiquitinization and proteolytic degradation of p105 by the 26S proteasome [52]. In the cotranslational mechanism, p50 and p105 can be generated from a single mRNA [53]. There is also evidence that the 20S proteasome constitutively processes p105 to p50 in a ubiquitin-independent manner [52]. The p50/p50 homodimer was originally considered to be a transcriptional repressor, but now there is substantial evidence that it can also induce gene transcription after a complex formation with the BCL3 nuclear protein, an atypical member of the I*κ*B family of proteins that is considered to be a coactivator of p50/p50 homodimers [34].

The link between NF-*κ*B activation by glutamate and the reduction of melatonin synthesis was evidenced with the use of PDTC, a NF-*κ*B translocation inhibitor [54]. PDTC prevented glutamate's inhibitory effect on melatonin synthesis. Taken together, the crucial presence of astrocytes for the inhibitory effect of glutamate, the exclusive activation of NF-*κ*B by glutamate in the same cell type, and the demonstration of NF-*κ*B's role in melatonin synthesis indicate that astrocytes under glutamate stimulation possibly release a factor that promotes a decrease in pinealocyte melatonin synthesis. TNF-*α* may be a good candidate. Indeed, TNF-R1, the TNF-*α* receptor that interacts with soluble TNF, is present in the pineal gland, reinforcing the importance of this signaling pathway [55]. Acting as an inhibitor of TACE to prevent TNF-*α* release, BB1101 was thus adequate in demonstrating the role played by TNF-*α*. In addition, the hypothesis that the p50/p50 homodimer may induce TNF-*α* gene expression and release needs to be clarified. 

In the CNS, astrocytes are known to release gliotransmitters such as glutamate, D-serine, and ATP [21]. Moreover, astrocytes can also synthesize and release proinflammatory cytokines in response to NF-*κ*B activation, such as TNF-*α* and interleukin-1, and induce gene expression of iNOS, resulting in nitric oxide formation [56]. In this work, we suggested that TNF-*α* is the factor that is released by astrocytes under glutamate stimulation because the TACE inhibitor antagonized glutamate's effects. Reinforcing this idea, recent preliminary data obtained in our laboratory with the L929 tumor cell lineage, which is sensitive to TNF-*α*, showed that astrocytes release TNF-*α* when stimulated by glutamate (data not shown). The literature states that TNF-*α* decreases the serotonin content and AANAT mRNA expression, resulting in the reduction of N-acetylserotonin [57, 58], the immediate precursor of melatonin synthesis. It is not clear whether TNF-*α* acts by itself or in combination with glutamate because there are glutamate receptors in the pinealocyte membrane [12, 15, 16, 18]. Recent studies have reported the control of hippocampal synapses by TNF-*α* due to the increased insertion of AMPA receptors in the surface of the neuronal soma [59]. The same effect could take place in the pineal gland, in which TNF-*α* released by the astrocytes in response to NF-*κ*B activation could increase the number of AMPA receptors in the pinealocyte membrane, resulting in the inhibition of melatonin synthesis. 

**Figure 9:**
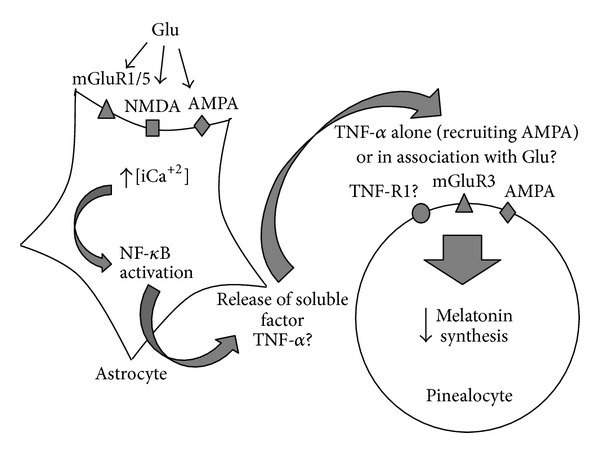
Diagram illustrating the putative paracrine interaction between pinealocytes and astrocytes in the modulation of pineal melatonin synthesis by glutamate. Glutamate (Glu) induces intracellular Ca^2+^ increase in astrocytes with the subsequent activation of NF*κ*B and release of a soluble factor, possibly TNF-*α*. This factor alone or in association with Glu promotes melatonin synthesis reduction in pinealocytes.

A model of the putative paracrine interactions between astrocytes and pinealocytes in the modulation of pineal melatonin synthesis by glutamate is shown in Figure 9.

## 5. Conclusions

In summary, according to our results, we propose that glutamate acts on glutamate receptors (AMPA, NMDA, and/or mGluR1/5) in astrocytes, increasing the intracellular calcium concentration, promoting NF-*κ*B activation, and driving the release of a soluble factor, possibly TNF-*α*, which acts independently or with glutamate in the pinealocytes, resulting in inhibition of melatonin synthesis.

## Supplementary Material

Supplementary Figure 1: Flow cytometer analysis of membrane integrity and DNA fragmentation of the pineal glands under glutamate influence.
Supplementary Table 1: Sequence of specific primers for rat glutamate receptors used in RT-PCR assays.

## Figures and Tables

**Table 1:** Summary of intracellular [Ca^2+^] changes in astrocytes and pinealocytes after stimulation.

Cell type/agents	Number of cells examined	[Ca^2+^]_*i*_ response %
Isolated astrocytes		
Glu 600 *μ*M	68	79
AMPA 50 *μ*M	97	80
NMDA 100 *μ*M	49	90
DHPG 50 *μ*M	80	74
Isolated pinealocytes		
Glu 600 *μ*M	66	80
AMPA 50 *μ*M	78	85
NMDA 100 *μ*M	53	90
DHPG 50 *μ*M	60	76

Intracellular [Ca^2+^] in fluo-4-loaded astrocytes and pinealocytes was measured as described in Section 2. The majority of the stimulated cells responded with an increase in intracellular [Ca^2+^] after the addition of either glutamate (600 *μ*M), ionotropic glutamatergic agonists (AMPA 50 *μ*M and NMDA 100 *μ*M), or a class I metabotropic glutamatergic agonist (DHPG 50 *μ*M).
