# Expression of Concern on “Modulation of Pineal Melatonin Synthesis by Glutamate Involves Paracrine Interactions between Pinealocytes and Astrocytes through NF‐*κ*B Activation”

**DOI:** 10.1155/bmri/9838035

**Published:** 2026-10-22

**Authors:** BioMed Research International

EXPRESSION OF CONCERN: D. Villela, V. F. Atherino, L. de Sá Lima, A. A. Moutinho, F. G. do Amaral, R. Peres, T. Martins de Lima, A. da S. Torrão, J. Cipolla‐Neto, C. Scavone, and S. C. Afeche, “Modulation of Pineal Melatonin Synthesis by Glutamate Involves Paracrine Interactions between Pinealocytes and Astrocytes through NF‐*κ*B Activation,” *BioMed Research International,* 2013, 618432, https://doi.org/10.1155/2013/618432

This Expression of Concern is for the above article, published online on 05 August 2013 in Wiley Online Library (wileyonlinelibrary.com), and has been issued by agreement between the Editor‐in‐Chief, Yoel Klug; and John Wiley & Sons Ltd. The Expression of Concern has been agreed due to concerns raised in Figure 6, some of which have been reported directly to the journal and also on PubPeer [1]. Specifically, there are unexpected repetitive elements within and between the Western blots shown in Figures 6a, 6b and 6c.

Due to the time elapsed since publication, the authors could not provide the original blots and therefore the results could not be independently verified. However, the Editorial Board notes that in the manuscript it is stated that these images were of the same probes, run on the similar extracts, and it is therefore plausible that they may appear similar. Therefore, the journal has decided to issue an Expression of Concern to inform and alert readers.
