# Supplementary material for: Modulation of Pineal Melatonin Synthesis by Glutamate Involves Paracrine Interactions between Pinealocytes and Astrocytes through NF-κB Activation
Source: Biomed Res Int. 2013 Aug 5;2013:618432. doi: 10.1155/2013/618432 (PMC3747608; doi:10.1155/2013/618432)
Supplement: Supplementary file 1 — Supplementary Figure 1: Flow cytometer analysis of membrane integrity and DNA fragmentation of the pineal glands under glutamate influence. Supplementary Table 1: Sequence of specific primers for rat glutamate receptors used in RT-PCR assays. [file 618432.f1.pdf]

Supplemental Table 1

Supplemental Table 1. Sequence of specific primers for rat glutamate receptors used in RT-PCR assays.

Primers/GenBank No	Size (bp)	Forward	Reverse
GluR1/NM_031608.1	203	5’-TTCTAGGTGCGGTTGTGGGTG-3’	5’-GGAGAACTGGGAACAGAATCGG-3’
GluR2/NM_017261.2	155	5’-ATTGTACACTACGATGATTC-3’	5’-CGGTGCACAGCATCGTCGGA-3’
GluR3/NM_032990.2	193	5’-TCTGCTCCCAGTTCTCCAGAGG-3’	5’-CCCAGAAGACTCAGAATGGCAC-3’
NR1/NM_017010.1	201	5’TACACTGCCAACTTCCGAGCTTTC-3’	5’-GCACAGTAACCAGGTTACTAGGGA-3’
mGluR1/NM_017011.1	79	5’-CCAACGGAGGGATCACAATAA-3’	5’-TCCAGCCTCAGCTTCAGGAA-3’
mGluR2/NM_001105711.1	198	5’-TGGCACAGGCAAGGAGACA-3’	5’-CGGTGGTTACAGCGCAATG-3’
mGluR3/NM_001105712.1	196	5’-TGCCTCTGAGGGTGACTATGG-3’	5’-ATGTTACGCAGCCGTGCTT-3’
mGluR5/NM_017012.1	165	5’-TTGTGGCAGCGACTGTCTGT-3’	5’-TCTTGGGAAAGGGTTTGATGA-3’
mGluR7/NM_031040.1	70	5’-CAGAAACGGAAGCGAAGCTT-3’	5’-GTTTGTGTGACAGCCTTGATGAC-3’

Supplemental Figure 1

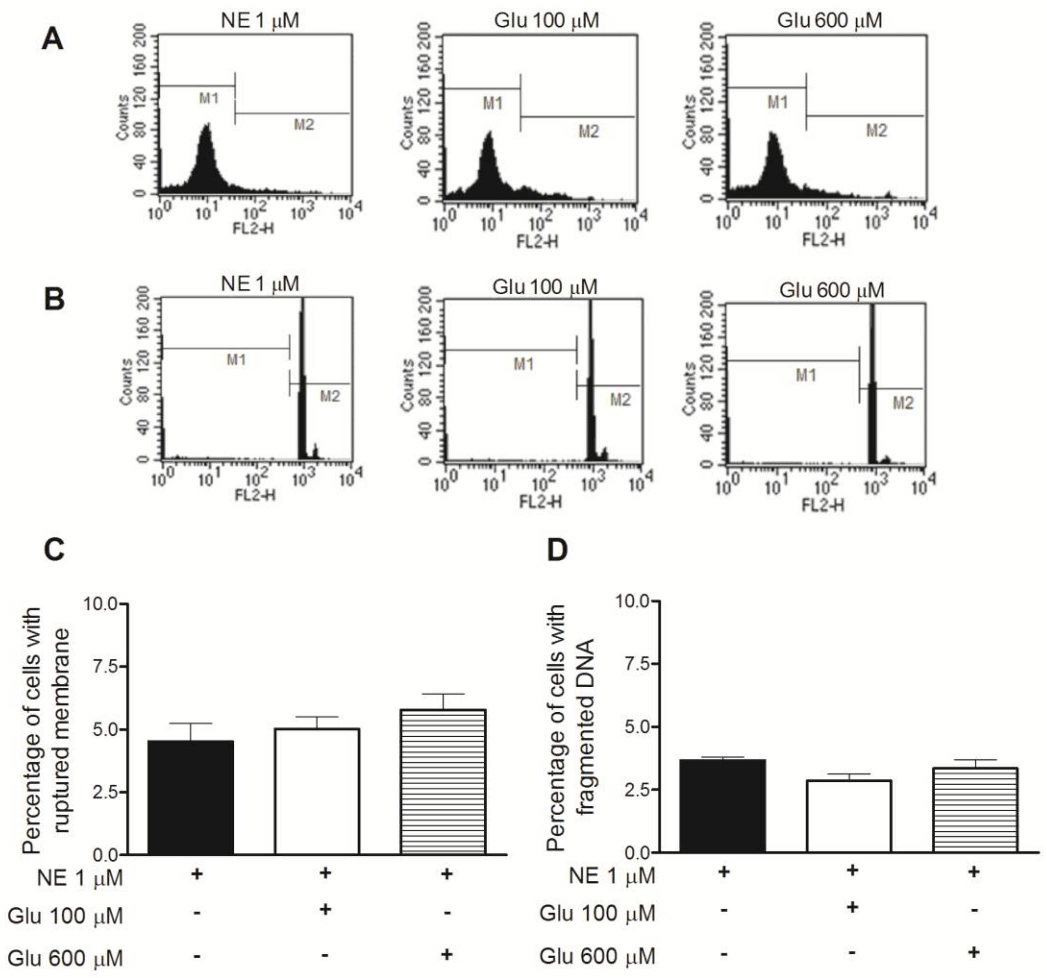

Supplemental Figure 1. Flow cytometric analysis of cell membrane integrity and DNA fragmentation. Pineal gland cells were treated with norepinephrine in the absence (NE) or presence of glutamate (Glu). Histograms show the intensity in the FL-2 channel, which corresponds to the propidium iodide emission wavelength. (A) Representative histograms of the membrane integrity analysis. (B) Representative histograms of the DNA fragmentation analysis. Percentage of rat pineal gland cells with ruptured membrane (C) and DNA fragmentation (D) after treatment with NE 1 µM and Glu 100 or 600 µM for 5 h. The values are presented as the mean ± SEM from three experiments.
